# Endoscopic ultrasound-guided choledochoduodenostomy as a life-saving salvage therapy for post-transplant acute biliary obstruction

**DOI:** 10.1055/a-2760-9303

**Published:** 2026-01-08

**Authors:** Yiran Song, Bixiong Zhang, Yue Sun, Yue Li

**Affiliations:** 1 372209 Department of Gastroenterology, The Affiliated Taizhou Peopleʼs Hospital of Nanjing Medical University, Taizhou School of Clinical Medicine, Nanjing Medical University, Taizhou, China; 2 89346 Endoscopy Center, Guangdong Provincial Peopleʼs Hospital, Guangdong Academy of Medical Sciences, Southern Medical University, Guangzhou, China

Endoscopic ultrasound-guided biliary drainage (EUS-BD) has become a recognized and effective alternative to conventional retrograde biliary drainage methods, such as endoscopic retrograde cholangiopancreatography (ERCP 1 2 ). While endoscopic ultrasound (EUS) is occasionally employed for diagnosing complications following liver transplantation, the feasibility of EUS-guided interventions for managing post-transplant complications has been documented in only a limited number of case reports 3 4 5 . We report the first case in which endoscopic ultrasound-guided choledochoduodenostomy (EUS-CDS) was successfully utilized as an emergency intervention in a liver transplant recipient with septic shock, following the failure of ERCP.

**Fig. 1:**
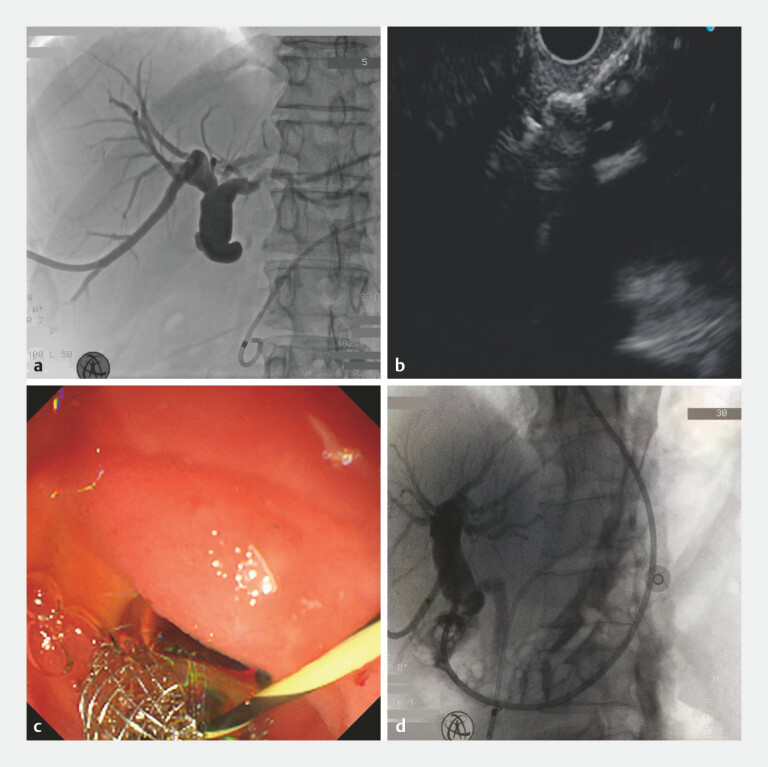
Endoscopic ultrasound-guided choledochoduodenostomy (EUS-CDS) for post-transplant biliary obstruction. **a** Endoscopic retrograde cholangiopancreatography (ERCP) failed to place a biliary stent; however, a pancreatic duct stent was placed successfully. **b** and **c** EUS-CDS with successful stent deployment. **d** A naso-biliary catheter was placed in the bile duct through the stent, and fluoroscopy confirmed the absence of leakage.

We report the case of a 61-year-old man who developed cholestatic jaundice and pruritus 1 year after orthotopic liver transplantation. Magnetic resonance cholangiopancreatography revealed a hilar biliary stricture. The patient underwent percutaneous transhepatic cholangiography drainage; however, catheter migration led to inadequate biliary drainage and persistent jaundice. Subsequent ERCP included the placement of a pancreatic duct stent but failed to relieve the biliary obstruction ( Fig. 1 **a** ). The patient rapidly progressed to septic shock following the procedure. After fluid resuscitation and vasopressor support, EUS-CDS was performed. An 8 mm × 6 mm self-expandable metal stent was deployed between the dilated common bile duct and the duodenal bulb, and an 8.5-Fr naso-biliary catheter was inserted into the bile duct to enable continuous irrigation ( Video 1 , Fig. 1 **b–d** ).

Procedure of endoscopic ultrasound-guided choledochoduodenostomy using a self-expandable metal stent.Video 1

Within 72 hours following the procedure, procalcitonin fell from >100 ng/mL to 12.7 µmol/L, and C-reactive protein decreased from 122.4 to 20.3 mg/L. Haemodynamic stability was restored, and vasopressors were weaned off. The patient was discharged home in stable condition 2 weeks postoperatively.

To the best of our knowledge, this case represents the first reported instance in which EUS-CDS can serve as a life-saving and minimally invasive rescue therapy for septic biliary obstruction when both conventional ERCP and percutaneous approaches have failed in a post-liver transplant patient. This finding may broaden the indications for EUS-BD in post-surgical patients with altered anatomy and life-threatening sepsis, establishing it as a viable emergent therapeutic option.

Endoscopy_UCTN_Code_TTT_1AS_2AD
